# Convalescent Plasma Reduces Endogenous Antibody Response in COVID-19: A Retrospective Cross-Sectional Study

**DOI:** 10.4274/tjh.galenos.2021.2021.0277

**Published:** 2021-12-07

**Authors:** Ahmet Omma, Abdulsamet Erden, Serdar Can Güven, İhsan Ateş, Orhan Küçükşahin

**Affiliations:** 1 Ministry of Health Ankara City Hospital, Department of Internal Medicine, Division of Rheumatology, Ankara, Turkey; 2 Ministry of Health Ankara City Hospital, Department of Internal Medicine, Ankara, Turkey; 3 Yıldırım Bayezıt University Faculty of Medicine, Department of Internal Medicine, Division of Rheumatology, Ankara, Turkey

**Keywords:** COVID-19, Convalescent plasma, Antibody, Immunity

## Abstract

**Objective:**

The aim of this study is to investigate post-COVID-19 antibody titers in patients who received convalescent plasma (CP) in addition to standard-of-care treatment.

**Materials and Methods:**

Hospitalized COVID-19 patients who received CP in addition to standard care were retrospectively investigated. Patients who received CP with a recorded total COVID-19 antibody test result after treatment were included. From among hospitalized COVID-19 patients who received only standard care with a recorded total COVID-19 antibody test result, a control group matched for age, gender, and comorbidities was formed. Total COVID-19 antibody index levels were compared.

**Results:**

Thirty-three CP recipients were enrolled in the study. The control group consisted of 34 age-, gender-, and comorbidity-matched standard-care patients. Median total COVID-19 antibody index levels were significantly reduced in the CP group.

**Conclusion:**

Although CP therapy may have benefits for disease outcome, its potential ability to hamper long-term immunity may be a problem.

## Abstract (tr)

**Amaç:**

Çalışmanın amacı tedavi sürecinde standart tedavilere ek olarak konvalesan plazma (KP) tedavisi uygulanan COVID-19 hastalarında takipte oluşan COVID-19 antikor düzeylerini incelemektir.

**Gereç ve Yöntemler:**

Yatarak tedavi alan COVID-19 hastaları içinde standart tedavilere ek olarak KP tedavisi alanlar retrospektif olarak incelenmiştir ve takipte COVID-19 antikor düzeyleri bakılmış olanlar çalışmaya dahil edilmiştir. Aynı zaman zarfında yatarak takip edilen, standart tedavi alan ve takipte COVID-19 antikor düzeyi bakılmış olan COVID-19 hastaları arasından yaş, cinsiyet ve komorbidite sıklığı eşleştirilmiş bir kontrol grubu oluşturulmuştur. İki grup arasında COVID-19 antikor düzeyleri karşılaştırılmıştır.

**Bulgular:**

KP tedavisi alan ve takipte antikor düzeyleri bakılmış olan 33 COVID-19 hastası çalışmaya dahil edildi. Kontrol grubunda 34 hasta mevcuttu. Median total COVID-19 antikor indeks düzeyleri standart yaklaşıma ek olarak KP alan grupta anlamlı olarak düşük saptandı.

**Sonuç:**

KP tedavisinin COVID-19 hastalarında sonuçlar üzerine olumlu etkileri görülmekle beraber hastalık sonrasında azalmış antikor yanıtı, uzun dönem bağışıklık üzerine olumsuz etkilerin bir göstergesi olabilir.

## Introduction

Convalescent plasma (CP) therapy is the transfusion of plasma containing polyclonal antiviral antibodies, obtained from recently ill donors who have fully recovered with sufficient antibody responses. Potential mechanisms of action for CP are virus neutralization, antibody-dependent virolysis, antibody-dependent antigen presentation, antibody-dependent cellular toxicity, and complement activation [1]. Enhancement of viral clearance is the foremost effect expected from CP therapy; therefore, administration in the early stages of the infection with high viral load and insufficient endogenous immunoglobulin (Ig) response may be more beneficial [2,3]. CP has previously been used for prophylaxis after contact in viral hepatitis, mumps, measles, and polio and is used as a therapeutic agent for influenza, severe acute respiratory syndrome (SARS), and Middle East respiratory syndrome (MERS) [4,5,6,7,8,9,10]. Likewise, the effectiveness of CP therapy with early administration has been demonstrated for coronavirus disease 2019 (COVID-19). In a retrospective cohort study based on the US national registry, the unadjusted mortality within 30 days after CP therapy was lower among patients who received a transfusion within 3 days after receiving a diagnosis of COVID-19 than among those who received a transfusion 4 or more days after the diagnosis [11]. In addition, Libster et al. [12] demonstrated reduced progression to severe respiratory disease with early CP administration. Contradictory results regarding the efficacy of CP in COVID-19 also exist [13].

Endogenous antibodies produced by the host in COVID-19 have protective effects against reinfection. Although early administration of CP seems to have beneficial effects on outcomes in COVID-19, it is unclear whether CP therapy alters the endogenous antibody production and hampers long-term humoral immunity against the virus. In this study, we aim to investigate post-COVID antibody titers in patients who received CP in addition to standard-of-care (SOC) treatment and compare them to those of patients who received only SOC treatment.

## Materials and Methods

### Study Design

This study was conducted as a single-center, retrospective, case-control study. Ethical approval of the study was obtained from the Ethics Committee of Ankara City Hospital.

### Patients

Hospitalized COVID-19 patients who received CP therapy in addition to the SOC approach from Ankara City Hospital’s Internal Medicine Inpatient Clinic between August 15 and December 31, 2020, were retrospectively investigated. The COVID-19 diagnosis was confirmed with the presence of a recorded positive SARS-CoV-2 real-time reverse transcription polymerase chain reaction (RT-PCR) test from a nasopharyngeal swab for every patient. Among patients with positive PCR results, subjects who received a total of at least 400 mL of CP obtained from donors (200-250 mL administered on two consecutive days or two alternate days) with recorded test results for total COVID-19 antibody against the S1 antigen (Siemens Atellica-IM Total [COV2T]) after PCR positivity were included in the study. Index values of COVID-19 total Ig over 1 are accepted as positive for this kit. Our center reported all values over 10 as >10; therefore, patients with Ig levels of >10 were recorded as having levels of 10. Data regarding demographics and comorbidities were recorded for all CP recipients. From among hospitalized COVID-19 patients who received only SOC treatment during the same period of time with recorded total COVID-19 antibody test results, an age-, gender-, and comorbidity-matched control group was formed.

### Interventions

The SOC approach comprised oxygen support, hydroxychloroquine, favipiravir, low-molecular-weight heparin, anticoagulants, and additional anti-inflammatory treatment when indicated in accordance with the COVID-19 guidelines of the Turkish Ministry of Health [14]. Likewise, indications for hospitalization, CP therapy administration, intubation, and discharge were also set in accordance with the Turkish Ministry of Health guidelines [14].

### Outcomes

Total COVID-19 antibody index levels and days from symptom onset at the time of COVID-19 antibody work-up were recorded for both groups.

### Statistical Analysis

Statistical analyses were performed using SPSS 22 (IBM Corp., Armonk, NY, USA). The normality of variables was investigated by Shapiro-Wilk test. Continuous variables were presented as median and interquartile range (IQR). Categorical variables were presented as number and percentage. The Mann-Whitney U test was used for comparison of continuous variables according to normality. For comparisons of categorical variables, the chi-square test was used. Values of p<0.05 were considered statistically significant.

## Results

Of 67 recipients of CP in addition to SOC treatment, 33 patients with total COVID-19 antibody test results were enrolled in this study. The control group consisted of 34 age-, gender-, and comorbidity-matched SOC patients with total COVID-19 antibody test results. Demographics, comorbid diseases, duration of symptoms at the time of COVID-19 antibody work-up, and total COVID-19 antibody titers are presented in Table 1. No significant differences were observed in the demographics or frequency of comorbid diseases between groups. Days from symptom onset at the time of antibody work-up was 28.5 (29.75) in the SOC group and 25 (12.5) in the SOC + CP group [median (IQR), p=0.292]. In the SOC + CP group, the interval between CP administration and antibody work-up was 21 (12.5) days [median (IQR)]. Median (IQR) total COVID-19 Ig levels were significantly reduced in the CP group (Table 1).

## Discussion

Our results demonstrate a significantly reduced total COVID-19 antibody response in CP recipients.

CP is obtained from recovered COVID-19 patients who have developed humoral immunity, containing neutralizing antibodies (NAbs) for severe acute respiratory syndrome coronavirus 2 (SARS-CoV-2) capable of pathogen clearance from peripheral circulation and pulmonary tissues [15]. NAbs particularly bind to the S1-receptor binding domain (S1-RBD) of the S protein, which binds to angiotensin-converting enzyme receptors, preventing the entrance of the virus into the cells. Furthermore, CP contains various IgG and IgM type non-neutralizing antibodies (non-NAbs), similar to fresh frozen plasma. In addition to enhancing viral clearance, both NAbs and non-NAbs have immunomodulatory effects via limiting immune complex formation and complement cascade activation [16,17]. Furthermore, both NAbs and non-NAbs in CP reduce innate immune activation and regulate the activation of these cells downstream of the Fcg receptors in B lymphocytes and antigen-presenting cells. Again, by regulating T lymphocyte interactions of these cells, they cause humoral tolerance against SARS-CoV-2 and reduce antibody formation [18]. Theoretically, CP administration during the active infection period may suppress the endogenous antibody response due to these aforementioned effects on both B lymphocytes and innate immunity. Our results have demonstrated reduced levels of COVID-19 antibodies in CP recipients after a median of 28.5 days from symptom onset in the SOC group and 25 days in the CP group. Since CP is generally administered within the first week of symptoms and the IgG half-life in circulation is 10 to 21 days, we may assume that even with the presence of considerable exogenous COVID-19 antibodies in circulation, the index COVID-19 antibody levels were still lower in the CP group, possibly indicating deterioration in endogenous antibody production [19].

CP therapy may enhance viral clearance and provide better disease outcomes, particularly when administered in early stages of infection [12,20]. However, an altered long-term humoral immunity due to suppression of endogenous antibody production may be speculated as a risk of CP therapy. Therefore, it should not be overlooked that after immunoglobulins in CP are metabolized by the host, an absence of immunological memory for SARS-CoV-2 may occur [21].

### Study Limitations

The small sample size and retrospective nature of this study were the major limitations. Disease severity at admission or onset of CP treatment was not evaluated. Furthermore, there may have been undetected variations in Ig levels of CP solutions since Ig levels were not measured. Finally, our center reported COVID-19 total Ig values over 10 as >10; therefore, patients with higher Ig levels were not evaluated precisely, which would have further reflected the altering effects of CP on endogenous Ig production. Nevertheless, to our best knowledge, this is the first study to evaluate effects of CP therapy on endogenous antibody production in COVID-19.

## Conclusion

In conclusion, although CP therapy may have benefits for disease outcomes, its potential to hamper long-term immunity and increase the risk of reinfection may be a problem, since the pandemic is still far from being under control globally.

## Figures and Tables

**Table 1:**
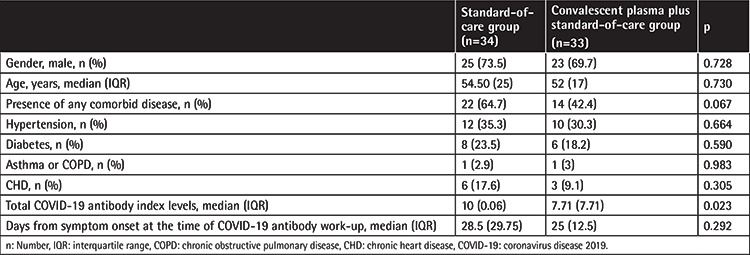
Demographics, frequency of comorbid diseases, days from symptom onset at the time of COVID-19 antibody work-up, and total COVID-19 antibody index levels in patient groups.
